# Transfusion-associated Hepatitis E, France

**DOI:** 10.3201/eid1304.061387

**Published:** 2007-04

**Authors:** Philippe Colson, Carole Coze, Pierre Gallian, Mireille Henry, Philippe De Micco, Catherine Tamalet

**Affiliations:** * Centre Hospitalier Régional Universitaire, Timone, Marseille, France; † Université de la Méditerranée, Marseille, France; ‡ Etablissement Français du Sang Alpes–Mediterranée, Marseille, France

**Keywords:** hepatitis E virus, transfusion, nosocomial infection, HEV RNA, immunosuppressive therapy, letter

**To the Editor:** Hepatitis E virus (HEV) is a leading cause of acute and fulminant hepatitis in developing countries (*1*). In industrialized countries, HEV seroprevalence rates of 0.4%–3% are common, and evidence is mounting that autochthonous transmission might account for a substantial proportion of infections (*1*–*3*). Whereas fecal-oral HEV transmission is preponderant in developing countries, other routes have been demonstrated in industrialized countries, among them consumption of pork or boar meat (*1*,*4*). Parenteral transmission was first suggested but not demonstrated (*5*,*6*). Because viruses cannot be fully inactivated in blood products, HEV recently emerged as a transfusion-transmitted pathogen, with 6 reported cases, including 3 in which comparative analysis of HEV sequences from blood donor and recipient was performed (*7*). We describe what is, to our knowledge, the first case in France and the first case worldwide involving a child as blood recipient.

A 7-year-old boy was examined in June 2006; he had erythematous-papulous skin and an increased level of alanine aminotransferase (ALT, 796 IU/L) of 1 week’s duration. From October 2005 to May 2006, he had received several courses of combined chemotherapy for a rhabdoid tumor in his kidney. Therapy included carboplatin, cyclophosphamide, etoposide, adriamycin, and vincristine, and, because of the tumor’s hematotoxicity, 22 transfusions of concentrated erythrocytes or platelets. Peak levels of ALT and bilirubin were reached 4 weeks after onset of hepatitis (2,001 IU/L and 49 μmol/L, respectively), and ALT levels returned to normal range (8–45 IU/L) 6 weeks later. During a follow-up examination, the boy’s prothrombin index remained >80%, and clinical signs mostly consisted of clinical jaundice. HEV diagnosis was established by detection of HEV antibodies in serum (EIAgen Kits, Adaltis Development Inc., Laval, Quebec, Canada) and HEV RNA with in-house assays. Other infectious or noninfectious causes of acute hepatitis were excluded. HEV immunoglobulin M (IgM) was weakly positive in June 2006 (optical density ratio = 1.6), then strongly positive the next month (ratio = 10.8); IgG remained negative. HEV RNA was detected from serum samples collected in June 2006 by an in-house real-time PCR that targeted the open reading frame 2 region of the HEV genome. Sequences of primers/probe are as follows: 5′-aattratttcgtcggcygg-3′; HevMrsRTRev: 5′acwgtcggctcgccattg-3′; HevMrsFam: 5′-FAM-actcycgccasgtygtctca-TAMRA-3′.

Serum samples taken from 12 U of packed erythroctyes that the child received, 1 U at a time, during the 3-months period before onset of hepatitis were tested for HEV RNA; 1 sample was positive. Concentrated erythrocytes (310 mL) from this positive blood donation were transfused to the child in May 2006, 4 weeks after collection and 6 weeks before acute hepatitis developed. HEV IgG and IgM antibodies were not detected in the blood donation, which indicates that the blood donation occurred during the prodromic phase of HEV disease. HEV nucleotide sequences from the blood donor and recipient were identical. Phylogenetic analysis showed that they clustered together and were closely related to genotype 3f, which is prevalent in Europe (Figure [*8*]).

**Figure:**
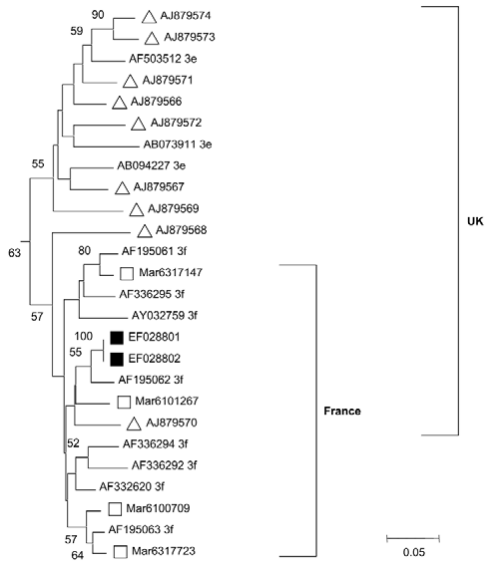
Outline of phylogenetic tree (complete figure available online, http://www.cdc.gov/EID/13/4/zzz-T.htm) constructed by the neighbor-joining method on the basis of partial nucleotide sequences of the open reading frame (ORF) 2 region of hepatitis E virus (HEV) genome obtained from the blood donor and the blood recipient, with sequences of other local HEV strains previously identified, and published HEV sequences. Local HEV sequences were obtained after reverse transcription–PCR amplification that used SuperScript One-Step RT-PCR System (Invitrogen Life Technologies with in-house protocols that used outer primers HevMrsFwd1: 5’-AATTATGCYCAGTAYCGRGTT-3’, 5663 nt (primer location is defined in reference to GenBank sequence accession no. NC_001434); HevMrsRev1: 5′-CCTTTRTCYTGCTGRGCATTCTC-3’, 6393, then inner primers HEVMrsFwd2 5’-GTWATGCTYTGCATACATGGCT-3’, 5,948 and HevMrs2Rev2: 5’-AGCCGACGAAATCAATTCTGT-3’, 6,295, to obtain a fragment of 347 bp. PCR fragments were purified then directly sequenced by using the inner primers and the Big Dye Terminator cycle sequencing kit version 1.1 on ABI Prism 3130 genetic analyzer (Applied Biosystems, Branchburg, NJ, USA). HEV genotype was determined by using phylogenetic analysis with a set of published HEV sequences (genotype and subtype are indicated with the GenBank accession no.) (*8*). Bootstrap values are indicated when >50% as a percentage obtained from 100 resamplings of the data. HEV sequences from the blood donor and recipient (indicated by black squares) have been submitted to GenBank (accession nos. EF028801 and EF028802); white squares indicate HEV sequences from other individuals with hepatitis E living in Marseille and its geographic area. Black inverted triangles indicate sequences involved in transfusion-transmitted hepatitis E cases in Japan (Japan A [*9*]); white inverted triangles indicate other Japanese HEV sequences from the same geographic area (Japan A [*9*]). White triangles indicate HEV sequences from the United Kingdom (*3*) that have an 80%–100% homology at the nucleotide level with those obtained by Boxall et al. (*7*). White circles indicate sequences from patients living in Hokkaido, Japan (Japan B; [*8*]); ORF 1 sequences from these strains were very similar to those characterized in a transfusion-transmitted hepatitis E case that occurred in Hokkaido.

The blood donor was a 24-year-old man. He did not travel outside metropolitan France for 8 months before donating blood. Anti-HEV IgG seroconversion was observed on a serum sample collected 24 weeks after blood donation, whereas anti-HEV IgM and serum HEV RNA tested negative. ALT levels were within normal values at the time of blood donation and 24 weeks later. No clinical signs were reported at any time. No other recipient received blood products from this donor.

Our data show that HEV was transmitted from 1 blood donor to the recipient child. On the basis of retrospective HEV RNA detection, 6 other transfusion-transmitted HEV infections, all involving adult blood recipients, have occurred in HEV-hyperendemic and industrialized countries (*7*). Transfusion-transmitted HEV strains belonged to different genotypes/subtypes that corresponded to those found in the same geographic areas (Figure).

In France, neither HEV antibodies nor HEV RNA are systematically tested in blood donors, and blood donations currently are not tested for ALT. In the absence of systematic HEV RNA testing, HEV diagnosis in blood donations may be hampered by HEV viremia before clinical onset and anti-HEV seroconversion, the possible short persistence or absence of HEV antibodies, and the high frequency of subclinical infections (*1*,*2*,*5*). On the basis of these data, improved knowledge of HEV epidemiology in the general population and in blood donors is needed in industrialized countries to guide HEV screening policy for blood products.

Another concern of transfusion-transmitted HEV infections is the potential severity of acute hepatitis E. Previously reported cases of contamination through transfusion and our case showed spontaneous recovery. However, although typically self-limited, hepatitis E is potentially life-threatening with mortality rates of 0.2%–4%, reaching 20% in pregnant women (*1*). Fatal outcome has also been described in children (*10*). In summary, HEV-contaminated blood donations are a challenge for diagnosis and virus safety of transfusion, including in industrialized countries.
